# Selfishness in Vehicular Delay-Tolerant Networks: A Review

**DOI:** 10.3390/s20103000

**Published:** 2020-05-25

**Authors:** Ghani-Ur Rehman, Anwar Ghani, Shad Muhammad, Madhusudan Singh, Dhananjay Singh

**Affiliations:** 1 Department of Computer Science and Software Engineering, International Islamic University, Islamabad 44000, Pakistan; Ghani84kk@gmail.com (G.-U.R.); anwar.ghani@iiu.edu.pk (A.G.); 2 Department of Computer Science and Bioinformatics, Khushal Khan Khattak University, Karak 27000, Pakistan; muhshad@gmail.com; 3 Endicott College of International Studies, Woosong University, Daejeon 300-718, Korea; 4 Department of Electronics Engineering, Hankuk University of Foreign Studies, Yongin 449-791, Korea

**Keywords:** Delay-Tolerant Networks, selfish behavior, individual selfishness, social selfishness, incentive schemes, cooperative communication

## Abstract

Various operational communication models are using Delay-Tolerant Network as a communication tool in recent times. In such a communication paradigm, sometimes there are disconnections and interferences as well as high delays like vehicle Ad hoc networks (VANETs). A new research mechanism, namely, the vehicle Delay-tolerant network (VDTN), is introduced due to several similar characteristics. The store-carry-forward mechanism in VDTNs is beneficial in forwarding the messages to the destination without end-to-end connectivity. To accomplish this task, the cooperation of nodes is needed to forward messages to the destination. However, we cannot be sure that all the nodes in the network will cooperate and contribute their computing resources for message forwarding without any reward. Furthermore, there are some selfish nodes in the network which may not cooperate to forward the messages, and are inclined to increase their own resources. This is one of the major challenges in VDTNs and incentive mechanisms are used as a major solution. This paper presents a detailed study of the recently proposed incentive schemes for VDTNs. This paper also gives some open challenges and future directions for interested researchers in the future.

## 1. Introduction

The internet has modernized the communication of people connected with one another. However, still, we find certain scenarios where the internet infrastructure is not available for communication. Some of these areas are disaster zones, big events like sports and other festivals, and areas where the internet is either too expensive or even not available. Such areas of populace need an alternative way of communication medium. Delay-Tolerant Networks (DTNs) [1,2,3,4,5,6,7,8,9,10] represent a network paradigm that is capable of overcoming the uncertainty in the fixed data networks by connecting mobile devices for communication.

Modern vehicles are equipped with GPS, digital maps, and other onboard units (OBUs), which enable these vehicles to communicate with one another (V2V) and vehicles to infrastructure (V2I) communication. This has made vehicular communications one of the active research areas. The introduction of short-range technologies in recent times for the wireless network is a key milestone. The two conventional communication technologies used in the vehicular networks are IEEE 802.11p-based Dedicated Short-Range Communication (DSRC) and vehicle-to-everything (C-V2X) [11]. The IEEE 802.11p-based DSRC has been on the market for nearly a decade. It has faced a number of challenges like unbounded channel access and lack of QoS issues for its distributed control. The other technology vehicle-to-everything (C-V2X) is new and has been in use in the past few years to overcome the issues in IEEE 802.11p technology. It is a cellular technology designed for transportation and connection of vehicles in the network. The cellular means in the technology as 4G LTE and 5G NR (New Radio) specifications and everything means varieties of users and vehicles. V2X will play an important role in the deployment of autonomous vehicles in the near future by delivering a predictable driving experience. However, both these technologies are in the evolution phase and are in deep discussion for further research. Another emerging and promising technology called fifth-generation network (5G) for a vehicle is an evolution of C-V2X, ensuring huge data requirements in high data traffic in-vehicle networks to overcome the major issues in the mainstream conventional communications. As a result, a number of up to date applications have been developed for vehicular ad hoc networks (VANET) [12,13,14,15,16] for comfortable journey services. The current 5G connected modern vehicles in South Korea is one of the prime examples of VANET [17,18].

In recent times, DTNs have been mainly used in scenarios where there are high delays and frequent disruptions in a network. One such scenario is VANET. Features like on-demand routing, self-organization, auto-configuration, and auto-recovery are distinct in the VANET. The topology of VANET can vary from a very dense network where there is a traffic jam, rush hour transport, etc. to a very sparse network where there is low traffic. The dense network has the greater capability of end-to-end multi-hop connectivity due to the availability of vehicles, while the end-to-end communication in the sparse network is low as there are fewer nodes available for communication. The sparse network of vehicular communication uses the store-carry and forward (SCF) mechanism and is the basis of DTNs. Such a variant of the vehicle network paradigm is called a Vehicle Delay-Tolerant Networks (VDTNs) [19,20,21,22,23]. VDTNs are novel way of vehicular communications where vehicles are used to communicate with each other as communication infrastructure for opportunistic communication in a network. VDTNs uses relay and store-carry-forward mechanisms for messages to be forwarded to the destination nodes without end-to-end connections in any DTN applications [24].

Although the two networks, VANET and VDTN [25,26], are independent and self-governed due to in-built node cooperation, some nodes may not contribute to the network performance by sharing resources. It is likely that some nodes in the network while using the resources of the network may not relay messages for other nodes; these nodes are termed selfish nodes. One solution is to grant incentives to the nodes to contribute in the network. Such incentives in the form of actual money or credit will stimulate the nodes for cooperation in the network. For example, the sender can give some incentives to the vehicle to store-carry and forward the sender data to the right destination [27,28].

**Figure 1:**
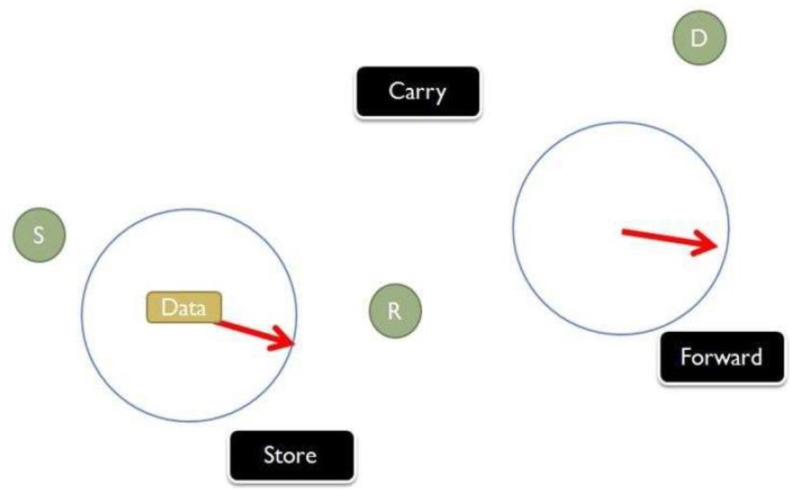
Store-carry-forwarding scenario in Vehicle Delay-Tolerant Networks (VDTNs).

Figure 1 shows a node collecting information at some source (S), the message is stored, and then it is forwarded to the destination location (D) by a passing vehicle. This type of opportunistic network is used to deliver location-based information like the gas station, parking, and any other road-related information to the display located at *D*. It can also be applied to a scenario where a message is forwarded to the gateway server located at *D* where there is no internet infrastructure.

The applications of the VDTN are to report traffic congestion, give updates on weather and roadside accidents, and many more. The traditional routing protocols DSR and AODV in such networks may not work properly due to fragmented nodes connectivity. Store and carry approach of the traditional routing forward the data to its destination regardless of the destination. The DTN protocols simulate the messages for an end-to-end delivery connectivity.

The selfish behavior shown by some of the nodes can degrade the performance of the VDTNs networks. It is seen that it can badly damage the VDTNs network performance due to selfish behavior. While using network resources the selfish nodes save their own resources like storage, space, and processing time [29,30,31]. This shows that selfish behavior is a challenging issue and needs to be addressed in a refined way. The two categories of nodes are normal and abnormal nodes. Normal nodes in a network cooperate with the neighbor nodes and actively participate in the network simulation, whereas abnormal nodes do not participate in the network activities and thus can degrade the performance of the network [32,33].

VDTNs represent a modern networking paradigm, in which vehicles act as communication infrastructure to communicate with one another. Some of the nodes may not forward messages to other nodes to save their resources. This is one of the challenging issues and different researchers have proposed different mechanisms to tackle the selfish behavior of the nodes in the VDTNs. They have used different criteria to stimulate the nodes for cooperation in the form of reputation, credit, and barter system. Some of the researchers have also used game-theoretic incentive schemes to encourage the nodes for cooperation. However, still, all the proposed mechanisms have some shortcomings and are addressed to date. This article presents a review of different incentive schemes. The main contributions of this review paper are as follows.

- Classified the selfish behavior of nodes into two different types, namely, collusion and non-cooperation.
- Classified different incentive schemes into four different types, namely, credit-based, reputation-based, barter-based system, and game-theoretic incentive schemes with advantages and disadvantages have been analyzed.
- The related work in different incentive techniques has been categorized and its contributions have been summarized.
- The selected techniques are compared on the basis of different parameters, namely, cost, approach, detection of nodes, scalability, applicability, detection accuracy, buffer consumption, etc.
- Future directions have been identified for the selfish behavior of nodes in VDTNs.

The rest of the paper is organized as follows. Section 2 provides background of selfishness and different types of selfish behavior. In Section 3, different papers are analyzed and classified according to the type of incentive techniques used to encourage node for cooperation within a network. Section 4 provides a comparison among different aspects of the selected documents and with a discussion related to some relevant findings for this review paper. The paper is finally concluded and future research findings are provided in Section 5.

## 2. Background

We see selfish people in our real world. Therefore, in the civilian DTNs called peopleNet [34,35] and other networks like packet switched networks [36], some of the connected nodes are not willing to forward packets to their neighbor nodes. These nodes are selfish and only use other nodes for their own interest and purpose. The selfishness is shown to reserve its own resources like energy, memory, and processing time [37,38,39,40], etc.

**Figure 2:**
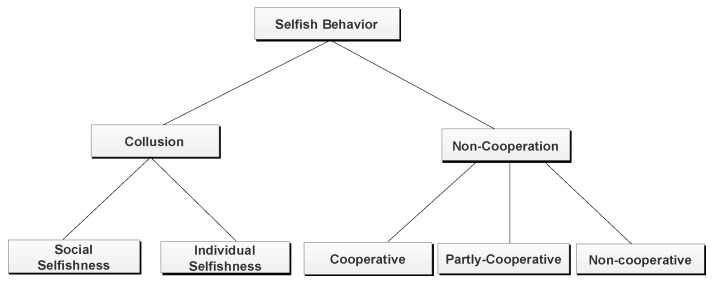
Types of selfish behavior.

In Ref. [41], the selfish behavior of nodes in the network has been classified into two categories: collusion and non-cooperation as shown in Figure 2 The collusion level of selfishness is either social selfishness or individual selfishness [42,43]. The social aspect means that nodes have a strong and weak relationship with fellow nodes. Social Selfish nodes improve their cooperation with other nodes in a network based on the social relationship to increase their social value. Social selfish nodes provide better services to nodes having a strong social relationship with them, and they exhibit weaker ties with weak social relationships, whereas individual selfish nodes show constant selfish behavior towards other nodes to increase their individual value. The social selfish nodes are not utterly different from the individual selfish nodes but are one of the general forms of it. In other words, when a node has no social relationship with the nodes outside, it becomes individual selfish. However, social selfishness has more meaning than the individual selfishness in most of the cases [44,45,46]. The other behavior of the node can be classified as non-cooperation. The non-cooperation nodes have three subcategories: cooperative, partly cooperative, and non-cooperative. The nodes with cooperative behavior are considerate and can store-carry and forward packets to the fellow nodes without constraints. Partly cooperative nodes forward packets to other nodes with certain restrictions, like message delivery direct to the destination. The non-cooperative nodes do not forward messages to other nodes received by them. Incentive-based mechanisms are used to manage the selfish behavior of the nodes in a network. Such a mechanism encourages selfish nodes to cooperate and share their resources in the network [47].

## 3. Methods

Some nodes are selfish in nature in the VDTN network. This issue has been addressed by many researchers. The selfish nodes in a network reduce the network performance by not cooperating with other nodes [48,49]. The incentive-based mechanism stimulates the nodes to cooperate in the network by sharing their resources in the network [50,51]. Some of the incentive-based mechanisms are credit-based, reputation-based, barter systems, and Game-theoretic based [52,53,54,55].

**Credit-Based Method:** This model motivates the nodes to cooperate and get a reward for it [56,57,58,59]. The concept of reward is that if any node forwards the message to other nodes, it gets an amount of credit as a reward. This reward can be used by the node for its own value later. It has a further two subcategories, namely, Message purse model and message trade model. In the message purse model, the source node is responsible for assigning credit to the intermediate nodes in participating in forwarding packets to the destination nodes. While in the message trade model, the destination node pays credit to the sender node for forwarding a message when it reaches the destination. As the source is not responsible for the payment, in the trade model, the intermediate nodes do trade with other nodes while sending the message to the next node or to the destination. Therefore, the intermediate nodes do earn some credit and the total cost of message forwarding is covered by the receiver, not the sender. This is the major drawback of the trade model as it creates network overloading. Thus, most of the credit-based mechanisms use the message purse model.

**Reputation-Based Method:** In a reputation-based mechanism, the node behavior is witnessed by other nodes. The node is assigned with a certain reputation based on its behavior [58]. The neighbor node gives its opinion to calculate the node reputation. The routing decision is taken by the value of the reputation: a lower reputed node has a low probability of next-hop selection in message forwarding [60,61,62,63,64,65,66,67].

**Barter-based Method:** Most of the uncooperative behavior of the nodes is tackled by the barter-based or tit-for-tat mechanism. It is very simple and is the most popular mechanism. In this strategy, every node forwards the messages for its neighbor as much as the neighbor forwards for it. The message has two categories: primary message and secondary message. If the mobile node is interested in the content of the message it is called a primary message, if not interested then it is called a secondary message. It is useful for the user to collect the messages even if not interested in the content, such a message can be exchanged for an interested message later on in the network [68,69,70].

**Game-Theoretic-Based Method:** Game theory is a mathematical framework model to understand the multi-user interaction and is applied to analyze the behavior of nodes in a network [71,72]. It can also be utilized to model the selfish behavior of nodes in a network for reward and punishment mechanisms. Table 1 presents the advantages and disadvantages of incentive methods discuss above.

### 3.1. Incentive Techniques

The selected publications encompass incentive techniques that can be categorized according to the type of incentive used (credit-based, reputation-based, barter-based, and Game-theoretic based). This classification can be seen in Table 2. In the next section, the contributions of each of the selected publications are summarized.

#### Credit-Based Schemes

In the literature, virtual credit is used as a solution for a credit-based mechanism. For example, Seregina et al. [73] found nodes participation as the main factor in DTN to relay messages. They proposed an incentive mechanism for the nodes who carry the source message to compensate for their cost. The incentives are different for every relay in this mechanism and are based on the meeting time with the source. The first one to relay the message gets the reward. The reward in this mechanism guarantees full cooperation. As the only node with the first relay will get the reward, the amount paid by the source node for delivery of messages to the destination lies between the first and last relay messages. It is shown that all three static settings get the same reward expected. We can say that, in other words, the expected reward that is paid to the relay by the source guarantees full cooperation irrespective of the information placed by the source, provided that the source does not keep the information it provides to the relay. The main disadvantage in this mechanism is that the reward assigned by the source to the relay nodes is the same. There is no reward distinction for full state information for any information delivered by the relay nodes.

The work of Park et al. [74] adopts a credit-based scheme integrated with blockchain-based cryptocurrency for cooperative VDTNs. One of the most important and famous cryptocurrencies is Bitcoin. It depends on a cryptography mechanism and distributed electronic payment method; this implementation does not depend on third-party trustees. Bitcoin overlay network or simply Bitcoin system is useful in unifying a practical credit-based system in VDTNs at a low cost. The main drawback of this system is of unlinkability because it is presumed every vehicle in a network has the single public key, any observer from outside can differentiate between two messages originated from the same user by tracing its fixed single public key. Gong et al. [75] has recommended social contribution-based protocol (SCR) for selfish nodes in vehicle network. The proposed system makes routing decision on the probability of delivering messages to the destination node on social contribution. The delivery probability is determined by the social ties between the sender and receiver nodes. The social contribution includes mutual contributions, community contributions, which stimulate the selfish nodes for cooperation in a network for better performance. Any node with stronger social ties and fewer social contributions has greater chances of next hope selection. The security procedures adopted for the payment process is the main drawback as some dishonest nodes might get payment.

The work of Dubey et al. [76] proposed a mechanism for selfish nodes to participate in the network for cooperation and get incentives. The proposed incentive-based mechanism helps in improving the mutual cooperation and reliability in vehicular networks. The vehicles in the network are encouraged by the reward it receives for transmitting private and public data. One routing protocol is used for data transmission in the network. In the VANETs paradigm, the performance of the network is improved by making all the nodes participate in the network. It means for better performance of a network, the nodes need to work in a coalition and motivate each other to get maximum benefits through rewarding schemes. Therefore, nodes involved in the process of sending data from source to destination form coalition. The coalition is in the form tree called forward tree. The main drawback of this proposed system is that incentive is not given according to the messages size. It effects the total transmission of the individual node and a bonus incentive is awarded to the last two nodes only.

Jiang et al. [77] recommended a novel credit-based incentive mechanism called congestion-aware incentive scheme (CBAIS) for DTNs. It has two functional components: a check and punishment mechanism, and a message acceptance selection mechanism. The first functional mechanism of check and punishment component check the congested node if it deliberately discards messages and punishes them. Any node who deliberately discard messages will be punished. However, it can also decide whether to accept more messages before its capacity is reached to full. The message acceptance selection mechanism in the proposed scheme is used to decide if more messages can be accepted for more credit-earning according to the congestion degree. A congested node can increase its acceptance message threshold for earning more credit. The main disadvantage of the proposed scheme is that each node needs a routing table for keeping the next hop node record.

The work of Xie et al. [78] has proposed a service priority-based incentive scheme (SIS). The proposed mechanism uses relaying as a service instead of credit as an incentive for stimulation of nodes in the network for cooperation. In the proposed scheme of SIS, a node with a higher bundle of relaying is given higher service priority and will receive a higher delivery ratio. To tackle the security attacks, three solutions are presented: signature chain, cooperation frequency statistics, and combination clearance. The main drawback with SIS is that it needs third-party supervision for trust manner.

Another credit-based scheme by Chen et al. [79] is a rewarding mechanism called Earliest Path singular Rewarding (EPSR). This scheme encourages the nodes to forward the message to its fellow nodes truthfully during every contact. As this scheme encourages the nodes to forward the messages to other nodes after every contact, the message delivers to its destination will vary different paths. The main theme of the EPSR is to reward the nodes in the earliest delivery path of the message delivery. Each node is rewarded on its contribution time in the earliest delivery path. The reward of the total successful messages are paid by the source node. The main drawback of the proposed scheme is that it does not consider all the elements of the selfish behavior of the nodes. It considers only memory, maximizing its own performance and some energy level. It is not considering selfish behavior elements like manipulation, self-centeredness, plotting scheme, etc.

The work of Ying et al. [80] recommended efficient incentive-compatible routing (ICRP), it has multiple copies of the message for two-hop DTNs and is using optimal sequential stopping rule [13] and algorithmic game theory approach [14]. In this proposed scheme, the optimal sequential stopping rule is used to determine the maximum reward relay node chosen by the source node, and the algorithmic game theory approach is used to calculate the reward value. It is shown that the relay node will get the maximum reward only when it honestly reports true contact value probability and routing performance metrics. This will stimulate the nodes in the network to participate actively in the process of relay node selection. To refine the strategy and guarantee true reporting Vickrey–Clarke–Groves (VCG) auction (second-price sealed-bid) is used for relay selection node. In this process, the bidder with the highest price wins, but the second highest bidder is rewarded. A node will not be rewarded if it refuses to forward messages to other nodes. It means the nodes will not get the services of the relay node in the future. This will alleviate the selfish behavior of the node in terms of discarding and refusing message forwarding. The limitation of the proposed scheme is that it uses the replication technique for multi-copy messages, and copies of successfully transmitted messages are still found on some of the intermediate nodes, minimizing the storage space.

### 3.2. Reputation-Based Schemes

Reputation-based strategies will work for our application, even though a large number of malicious nodes are involved. Moreover, this kind of strategy incorrectly considers intermediate node collaboration as selfish behavior resulting in decreases in the delivery probability of the generated messages.

The work of Dias et al. [81] has recommended CWS, a cooperative watchdog system to identify the selfish behavior of nodes in the network. The main task of the CWS is to assign a reputation to each node in the network. Therefore, when the nodes in the network come in contact, CWS updates its reputation score. Three-module classification, neighbor’s evaluation, and decision are used to calculate reputation score. The classification module categorizes the type of node according to its reputation score. It is also used to calculate the cooperation value of the node. A cooperative value is used by the decision module to reward or punish the node in the network. The neighbor’s evaluation module is used to evaluate the node’s reputation on the network. The neighbor’s opinion is used to accomplish the neighbor’s evaluation module.

After the end of each contact opportunity, the decision module updates the reputation score value based on information from other modules. This approach of CWS is used to organize, observe, and then act accordingly against such nodes. The CWS sends an alarm to all the neighbors when it detects a selfish node, this information is spread over the network. This alarm will also inform the cooperative nodes about new selfish nodes in the network. The main drawback of the proposed scheme is that the performance of the watchdog system decreases as the number of selfish nodes increases.

Rehman et al. [82] worked on incentive and punishment of selfishness in the network. They recommended an incentive and punishment scheme for the selfish nodes in the network. The main objective of the scheme is to encourage the nodes to participate in the network and cooperate with the nodes. It is very important to participate in the network, as IoV has lower access to the roadside units and other objects. In IoV, the single cluster of the connected nodes has uni-direction of movement so the system responsible for monitoring is very active. Each cluster has three heads elected after the election process: cluster Head, Auxiliary Cluster Head, and Incentive Head. The heads election is based on weight, cooperation, and the number of votes. The node having higher weight, cooperation, and a greater number of votes is elected as Cluster Head (CH). The node having second and third highest weight, cooperation, and a greater number of votes are elected as ACH and IH, respectively. The elected heads CH, ACH, and IH select monitoring nodes to monitor the behavior of nodes in the network. The nodes with selfish behavior are punished and nodes with cooperative behavior are rewarded with incentives in the form of reputation. The social technique is used to implement IPS in IoV to encourage selfish nodes to cooperate in network operations. This proposed scheme helps in omitting the selfish behavior of the node through punishment, and it encourages cooperative behavior by rewarding incentive in the form of reputation. The major drawback of the proposed scheme is that the monitoring node can also be selfish.

Dias et al. [83] have recommended a decentralized system that has the capability to detect the non-cooperative nodes in a network. Each node in the network has a reputation score. This reputation score defines the percentage of resources the node can share with other nodes. The reputation score of each node is equal to 50 at the beginning of network activities. This value may change between 0 and 100. To update the reputation score of an individual node and disseminate it across the network, CWS uses an out of band signaling approach. Nodes in the network share their information like dropped packets, delivered bundles, number of relayed packets, etc. with other nodes to illustrate their performance in the last contact opportunities. This information exchanged is used by the nodes to gauge each other and is stored in the reputation table of each node. CWS also collects and stores the same information about the nodes to evaluate their performance. The main issue with the proposed scheme is that the delay of bundle delivery increased as the cooperation threshold value decreased.

The work of Lin et al. [84] is a novel reputation-based scheme to estimate the node behavior. Every node in the intermittent network stores and forwards the messages. However, due to limited resources, the selfish node may not take responsibility for delivering the messages. Sometimes it is difficult to distinguish between selfish behavior and the inability of the node to deliver a message. This proposed scheme combines the selfish behavior and inability behavior of the node to estimate the reputation of the node. Results have shown that the proposed mechanism is effective and widely accepted and applied. The higher mobility of nodes improves the frequency of the contact opportunities; this helps the possibility of encountering the destination nodes. The threshold reputation value is beneficial in improving the node performance; its load, speed, and density. The node’s reputation value conceptually shows its behavior in the network. The information extracted from the network exactly evaluates the node’s behavior. For example, any relay node drops messages, but if the available space is enough, this dropping behavior is not reasonable. Similarly, if a node delivers only its messages with high priority then it may be a selfish behavior. Therefore, if more information about the delivery is collected better flexible approach could be designed. The main issue in the proposed scheme is that of a threshold value. Some of the nodes with a higher threshold value could be expelled as they are not qualified for the network activities.

Dias et al. [85] proposed a system that reduces the impact of selfish nodes in VDTNs. They proposed an optimized reputation score which is calculated through node performance in VDTNs. The reputation increases with each successful delivery of bundle, and it decreases if it drops the bundle at least once without sending it. In this way, node reputation score is used to add nodes to the blacklist and also used to set a threshold value of node cooperation. The node threshold value determines the resources it shares with other nodes in the network. The major limitation of the proposed scheme is that the bundle delivery ratio decreases with an increase in selfish nodes.

Kou et al. [86] proposed to detect and punish selfishness (DPS) algorithm. It is an incentive cooperation mechanism based on the punishment paradigm. DPS motivates nodes to cooperate and punishing measures are taken when there is selfish behavior. The communication among nodes in the network is improved by this reward and punishment mechanism. The major limitation of the proposed scheme is that each node in the network has reputation value and it also needs to know about the neighbor node behavior. Therefore, every node required its own memory to keep the record of reputation table.

Magaia et al. [87] proposed a novel reputation-based scheme for information-centric vehicular applications. This scheme is powered by machine learning and an artificial immune system (AIS) called ReFIoV. Each node learns newly detected data about node behavior and is made available for classification by using Bayesian learning. A K-algorithm is used to take recommendations from other nodes even if they show misbehavior. The AIS improves the detection of misbehavior nodes. This scheme can also be applied in a distributed manner as each node in the network decides its interaction with other nodes. The drawback of the proposed system is that as the number of selfish nodes increases, the possibility of delivery is decreased due to the fewer incentives for forwarding.

The work of Moreira et al. [88] is based on a centralized reputation system. A trust management system is needed in VANETs to keep the behavior record of the vehicles. In this proposed scheme, the well-behaved vehicle has two components of opportunistic forwarding messages: a reputation for the creation of messages and forwarding of messages. Every vehicle in the network can forward messages at any given time; the type of messages forwarded in every transaction may require the reputation score of the vehicle. Therefore, whether the receiver vehicle accepts the message is based on the reputation score of the sender vehicle that created and forwarded the message. The main drawback of the proposed system is that the exchange of information among vehicles may lead to leakage of the drivers private information.

### 3.3. Barter-Based Schemes

In the barter system, the nodes in contact share the equal amount of information. It is also called the tit-for-tat scheme.

The work of Liu et al. [89] recommended the concept of the node to community barter (node-to-com). This system proposed the bartering of services between nodes and communities in terms of message forwarding. The community gets benefits from the node as it forwards services to the community and the community contributes to the node when the node gets some forwarding help from it. In this way, the contributions of the node to the community and vice versa is balanced. The Node-to-com system improves message exchange probabilities by expanding the bartering range. The proposed com-BIS system is intended for routing having only one destination. By expanding the range of exchange from node-to-node to node to com barter, it improves the exchanging probabilities. The major drawback of the system is its overhead comparing with other competitors.

Zhou et al. [90] is an incentive-driven and freshness-aware public/sub content dissemination scheme. It is called ConDis and deals with the selfish behavior of nodes in OppNets. It employs the Tit-for-Tat (TFT) paradigm in OppNets to deal with the selfish nature of the nodes. This technique is based on the exchange protocol when nodes come in contact with one another. The content utility has two values direct subscribed and indirect subscribed; this is the basis for the order of exchange during each contact. Each node objective is to maximize the content inventory recorded in their memory buffer. It is observed in the proposed scheme that the system performance degrades as the number of channels increases.

Buttyan et al. [91] proposed a barter system that discourages the selfish behavior of the nodes in the network. This proposed mechanism uses the game-theoretic model, which stimulates the nodes for cooperation in the network. The cooperation among mobile nodes is based on the barter principle. It is observed that when two mobile nodes come in contact with each other, they exchange the description of messages they currently hold, and then they share the subsets of messages they agreed upon in. To improve the fairness in the proposed system, the selected subsets of messages must be of the same size and be shared message by message in certain preference orders. The exchange is disrupted if any node shows selfish behavior or cheats, this way the honest party avoids major disadvantage (the honest party has at most one less downloaded messages than the selfish and misbehaved party). It is noted that it is up to the mobile nodes to decide which messages they want to exchange and share with each other. The node shows selfish behavior by downloading only those messages of their concern and interest. However, this selfish behavior of the node will not be helpful for a long time. The main idea is that a message may not be of interest to node A, but it may be interesting for another node B, and node A may use this message to download a message from node B, which is certainly interesting for A. It is noted that a message which is secondary for a node may still be valuable for barter and worth to download and store it. Therefore, the secondary messages can be an asset for barter to new messages later. The performance of the system degrades highly with the increase in the selfish nodes in terms of delivery of messages. Sometimes the message is delivered with high latency and hence can degrade the system performance.

### 3.4. Game-Theoretic Schemes

This section discusses some game-theoretic schemes that are also used to motivate and encourage selfish nodes to cooperate with other nodes within a network.

Chen et al. [92] describe a novel game-theoretic incentive approach that gives cooperative incentives to the nodes. This technique also encourages the nodes to participate in the network activities by following defined rules for desired network performance. This proposed scheme identifies two aspects of node cooperation in the network: node in the network should participate in messages forwarding and it should also store messages as desired for the network performance. The limitation of the proposed scheme is that the motivation of nodes to forward messages in different sequences cannot be achieved for different performance objectives.

Yin et al. [93] describe a game-theoretic incentive scheme to motivate nodes to cooperate in forwarding messages. The proposed scheme is based on the pay-for-gain approach, it uses loan–credit theory to stimulate the nodes interest to forward messages in the network. The proposed scheme examined the equilibrium condition of the node to maximize node interest in the network. The main drawback of this scheme is that it is applied to a network where topology does not change and communication can not be disconnected.

## 4. Discussion

This section discusses the related work and summarizes the incentive techniques based on the proposed parameters. The related work is discussed and investigated with the following parameters.

**Incentive Schemes:** There are various techniques to evaluate the selfish behavior of the nodes in a network. This parameter identifies incentive schemes like credit-based, reputation-based, barter-based, and game-theoretic based.

**Cost:** To detect the selfish behavior of the nodes in the network, some algorithms are costly in terms of deploying an extra node to detect selfish nodes. To process the algorithm computations, both sender and receiver need computational power as well. There are two levels of cost taken here: High and Low. The level High is denoted by H and Low is denoted by L. The cost can be calculated as follows.

Cost = ProcessingCost + ExtraNodeCost

**Approach:** This parameter identifies the algorithm being used: detective or preventive.

**Detected Nodes:** There are various types of selfish nodes in a network. Some of the nodes are selfish, others are socially selfish or can be malicious nodes.

**Scalability:** Scalability is the ability of the network to adapt to future growth in a number of hops or nodes. Two levels are used for scalability: Yes or No.

**Detection Accuracy:** There are various algorithms that deal with the detection of selfish nodes in VDTNs. The ration of false positive to false negative is high and it is dreadful for VDTNs. This article considers three levels: low, average, and good based on these annotations.

**Buffer Consumption:** The buffer of the nodes sometimes overflows due to overloaded stored history and other information by some of the proposed algorithm. This can decrease the packet delivery ratio and performance of the network. three levels of detection algorithms are considered: high, medium, and low.

**Applicability:** This is one of the important parameters because some proposed schemes are applicable to DTNs and others are applicable to VDTNs.

Table 3 provides a detailed comparison of the different selected documents.

**Advantages:** Every technique has some advantages to it, this parameter deals with the advantages in the schemes discussed in the literature.

**Disadvantages:** Every technique has some limitations to it, this parameter deals with limitations and shortcomings in the selected schemes discussed in the literature.

The advantages and disadvantages parameters of the selected documents are discussed in Table 4.

## 5. Conclusions and Future Research Directions

This paper is organized to suggest and provide insight into the contemporary developments in the field of DTNs, focusing on the selfish behavior of the nodes. Stimulating nodes in the network for participation could improve its performance. All the nodes in the network try their best to forward the messages to other nodes. However, sometimes the selfish nature or the inability of a node to forward the messages can degrade the performance of the network. The selfish behavior of nodes is a major challenge in VDTNs where the nodes do not take responsibility to forward the messages to save its resources, such as computational power, memory, etc. Various incentive schemes are proposed to address the issue of selfishness in the VDTNs to improve its performance. These schemes award incentives to the nodes for active participation in the network activities. Four main types of incentive schemes, namely, credit-based, reputation-based, barter-based, and game-theoretic-based are used to tackle the issue of selfishness in VDTNs. These schemes encourage selfish nodes to cooperate in network activities. However, still, some of the challenges in VDTNs are addressed and discussed in the literature to grip the selfish behavior of the nodes. It is, however, hoped that our article on the detecting and omitting of selfish nodes through different incentive schemes would encourage the researchers in selfishness related topics in VDTNs. This article highlights the following different research areas and future directions in VDTNs.

- More parameters should be analyzed in the study in addition to cost, buffer consumption, and detection accuracy.
- The fair exchange principle greatly improves the cooperation ratio accomplished by the barter-based system. However, sometimes it is very challenging for the connected nodes to reach the level of complete fair exchange principle. Therefore, it is very tough for the connected nodes to make an arrangement during rounds of bargaining. This incomplete agreement between two nodes could waste the valuable resources of the nodes like power, energy, and bandwidth. In what manner the round bargaining problem should be tackled remains an ongoing problem.
- There are always selfish and malicious nodes in the network. The main challenge is not to detect the selfish behavior of the nodes but to identify all those packets which are malicious in nature.
- The preventive algorithm should be designed to prevent the nodes from overusing its limited resources.
- Security impeachment is also a very serious issue and we should consider it. Such security threats have a negative impact on these networks and some solutions must be presented to reduce its negative impact.
- Selfish behavior such as maximizing its own performance, saving buffer, CPU time, and memory are discussed in the literature. However, still there are types of selfish behavior such as plotting and scheming, self-centeredness, and manipulation are completely ignored.

## Figures and Tables

**Table 1:** Advantages and disadvantages of incentive methods.

Methods	Advantages	Disadvantages
Credit-Based	Motivate nodes in the network for cooperation through reward	It is not suitable for scenarios where there is a high probability of selfish nodes
Reputation-Based	Even useful in an environment where the majority of the nodes in the network have selfish behavior	Consider intermediate node cooperation to be selfish behavior
Barter-Based	Exchange of the same amount of message needed	If the node has not sufficient messages to exchange, then it cannot deliver messages to another node even when there is a connection with the destination node hence degrading the routing performance
Game-Theoretic-Based	The bargaining possibility increases with an increase in the number of nodes	The cost of game-theoretic approaches are high as it uses more resources

**Table 2:** Techniques classification according to incentive types.

Credit-Based	Reputation-Based	Barter-Based	Game-Theoretic Based
Seregina et al. [73]	Dias et al. [81]	Liu et al. [89]	Chen et al. [92]
Park et al. [74]	Rehman et al. [82]	Zhou et al. [90]	Yin et al. [93]
Gong et al. [75]	Dias e8 al. [83]	Buttyan et al. [91]	
Dubey et al. [76]	Lin et al. [84]		
Jiang et al. [77]	Dias et al. [85]		
Xie et al. [78]	Kou et al. [86]		
Chen et al. [79]	Magaia et al. [87]		
Ying et al. [80]	Moreira et al. [88]		

**Table 3:** A comparison among different aspects of the selected documents.

Article	Incentive Schemes	Cost	Approach	Detected Nodes	Scalability	Detection Accuracy	Buffer Consumption	Applicability
Seregina et al. [73]	Credit	H	Detective	Selfish	Yes	Average	Medium	DTN
Park et al. [74]	Credit	H	Detective	Selfish	Yes	Good	Low	VDTN
Gong et al. [75]	Credit	H	Detective	Social Selfish	No	Average	Medium	VDTN
Dubey et al. [76]	Credit	H	Detective	Selfish	Yes	Low	High	VDTN
Jiang et al. [77]	Credit	H	Preventive	Selfish	No	Average	High	DTN
Xie et al. [78]	Credit	H	Detective	Selfish	No	Average	Medium	DTN
Chen et al. [79]	Credit	H	Detective	Malicious	Yes	Average	Medium	DTN
Ying et al. [80]	Credit	H	Preventive	Malicious	Yes	Good	Low	DTN
Dias et al. [81]	Reputation	H/L	Detective	Selfish	Yes	Average	High	VDTN
Rehman et al. [82]	Reputation	H/L	Preventive	Selfish	Yes	Average	Medium	VDTN
Dias et al. [83]	Reputation	H/L	Detective	Selfish	Yes	Average	Medium	VDTN
Lin et al. [84]	Reputation	H/L	Preventive	Selfish	Yes	Average	High	DTN
Dias et al. [85]	Reputation	H/L	Detective	Social Selfish	Yes	Good	Medium	VDTN
Kou et al. [86]	Reputation	H/L	Preventive	Selfish	Yes	Average	High	VDTN
Magaia et al. [87]	Reputation	H/L	Preventive	Selfish	No	Average	High	VDTN
Moreira et al. [88]	Reputation	H/L	Detective	Selfish	Yes	Good	Medium	VDTN
Liu et al. [89]	Barter	L	Detective	Selfish	No	Low	Low	DTN
Zhou et al. [90]	Barter	L	Detective	Selfish	No	Low	Low	DTN
Buttyan et al. [91]	Barter	L	Detective	Selfish	Yes	Low	Low	DTN
Chen et al. [92]	Game	H	Detective	Selfish	Yes	Average	Medium	DTN
Yin et al. [93]	Game	L	Detective	Selfish	Yes	Low	Low	DTN

**Table 4:** Advantages and disadvantages of the selected documents.

Article	Advantages	Disadvantages
Seregina et al. [73]	Use of adaptive strategy: The source chooses to provide the relay with complete information, partial information or even no information at the time it reaches it, depending on the contact times with that relay	The reward assigned by the source to the relay nodes is the same. There is no reward distinction for full state information to no information delivered by the relay nodes.
Park et al. [74]	Usage of the unique public key procedure to improve vehicle interactions privacy and reward transactions.	It does not satisfy unlinkability requirement
Gong et al. [75]	Makes forwarding decisions on the basis of the delivery probability and the social involvement of the relay node	No proper security measures adopted in the payment process
Dubey et al. [76]	Allocate rewards for relay nodes to minimize selfishness and increase network coalition.	Bonus is given to the last two nodes only
Jiang et al. [77]	Monitoring system to discourage overloaded nodes from intentionally discarding messages	Each node needs routing table for indicating next hop
Xie et al. [78]	Optimize network bandwidth and the overall delivery ratio for bundles Uses a service priority to encourage cooperation among nodes	Uses third-party supervision for trust manner
Chen et al. [79]	Prevent typically manipulative behavior of selfish nodes	Ignore some of the selfish behavior such as plotting and scheming, manipulation and self-centered etc.
Ying et al. [80]	Better defense against attacks by forgers Forwarding nodes are selected according to the optimal sequential stopping rule	Multi-copy messages replication
Dias et al. [81]	Watchdog mechanism aiming at detecting and avoiding selfish nodes in order to minimize their influence on cooperative node performance	The performance decreased with increase in selfish nodes
Rehman et al. [82]	Solving Weight tie problem by using cooperation criteria	Monitoring node can also be selfish but it only monitors the behaviors of the selfish node
Dias et al. [83]	Control the number of resources exchanged with several other nodes so that the credibility of nodes is not compromised	When threshold value of the cooperation decreased then the delay of bundle delivery gets increased
Lin et al. [84]	Requires minimal computing power and a minimal amount of memory space for storing private informations	Nodes may be expelled from the network due to high reputational threshold
Dias et al. [85]	Uses scalable reputation system variant so as to prevent selfish node form behaving selfishly	Message delivery is decreased when the number of selfish nodes are increases
Kou et al. [86]	Selfish nodes are detected and their behaviors are recorded	Every node needs its own local memory for saving reputation table
Magaia et al. [87]	Very limited number of selfish nodes identified incorrectly	Every node needs its own local memory for saving reputation table
Moreira et al. [88]	Vehicles received confirmation on the behavioral variables for message generation and forwarding	Lack of privacy
Liu et al. [89]	Useful for improving the probability of exchanging messages	It generates high overhead
Zhou et al. [90]	Contents utility decides the exchange order in each contact	When the number of channels are increases then its performance is degrade
Buttyan et al. [91]	Users receive and transmit messages even when they are not involved in them and thus by reducing selfishness	High latency issue
Chen et al. [92]	Performance of the network improves with a high hit rate in messages forwarding and storing	Motivation of nodes to forward messages in different sequences cannot be achieved for different performance objectives
Yin et al. [93]	It is useful to stimulate cooperation among selfish nodes in the network	applied to networks where topology does not change and communication can not be disconnected
